# Inquiries into the Healthy and Diseased Appearances of the Mucous Membrane of the Stomach and Intestines

**Published:** 1828-02

**Authors:** W. E. Horner

**Affiliations:** adjunct Professor of Anatomy in the University of Pennsylvania.


### STOMACH AND INTESTINES.

*Inquiries into the Healthy and Diseased Appearances of the Mucous Membrane of the Stomach and Intestines.* By W. E. Horner, M.D. adjunct Professor of Anatomy in the University of Pennsylvania. (Condensed from the *American Journal of the Medical Sciences.*)

The want of some standard whereby the healthy and diseased appearances of the stomach and bowels may be distinguished, has been felt, perhaps, by all who have engaged in researches into the pathology of these organs. Though much has been written on the subject, yet the vagueness of the language used by authors, and more especially the great inaccuracy with which they employ the terms representing colours, have opened one of the most extensive fields for disputation in pathology.

In the numerous post-mortem examinations which I have made, both for myself and my professional friends, I have frequently found that the most opposite conclusions have been drawn from identical appearances in the organs under consideration; and there existed no available experience or authority, by which the correctness of these conclusions could be tested, and the truth determined. These circumstances induced me to institute a series of observations on the gastro-intestinal mucous membrane; and, by coincidences entirely accidental, I have been so fortunate as to obtain in a few months a description of information, that, in the ordinary current of events, might not have been acquired in years.

Three unsettled points present themselves in this inquiry: —1st. What is the healthy condition and appearance of the gastro-intestinal mucous membrane? 2d. What is its appearance in congestion from the agonies of dying? 3d. What is its appearance in genuine red inflammation?

It is necessary, in the commencement of this discussion, to settle the signification of the terms used: this I shall do, not in an arbitrary manner, but according to the meaning attached to them in conversation by some of the most sagacious and intelligent physicians of our city. I am forced to depend upon the inferences of conversation, because the definitions of Dictionaries do not decide; indeed, the terms are there used promiscuously. Thus, for example, Parr says that congestion is a swelling that gradually arises and slowly ripens, in opposition to that defluxion which is quickly formed and terminated. In the Dictionnaire des Sciences Medicales, congestion is defined to be a humoral collection which is formed slowly in some part of the body, increases gradually, and finishes by an intumescence of a variable size; the fluids which form it may be blood, serosity, pus, fat, bile, urine, —in one word, any of the fluids belonging to the body. It is thus evident that Parr alludes to the sub-inflammation of modern pathology, and that the other has no specific meaning. I now therefore state, that by *congestion* I mean an accumulation of red blood in any part or organ of the body, without irritation or mechanical violence; and by *red inflammation*, the accumulation of red blood which follows any local irritation.

### I. *Of the Natural Colour of the Mucous Membranes.*

When an animal is bled to death, the stomach being empty, the mucous membrane of the latter, and of the intestines, is of a yellowish pearl colour, presenting at a short distance the lightest possible tint of pink; and few or no marks of blood exists, even in the large vessels under the peritoneal coat.

April, 1827.—At a butchery in Spring Garden, a sheep, which had fasted for twenty-four hours, was slaughtered in the usual way, by cutting the throat, and immediately afterwards dividing the spinal marrow in front between the occiput and first vertebra. As soon as life was extinguished, we examined the abdomen. The internal membrane of the stomach was of a pearl colour, slightly yellow, approaching to what artists call a light tint of bright brown; and the intestines were almost exactly like the stomach. Neither stomach nor intestines exhibited their blood-vessels, even of the largest size, except very faintly; and there were no red patches in either.

If an animal be killed with a full stomach, by puncture of the medulla spinalis at its commencement, the mucous coat of the stomach will retain on the surface, where the food was in contact with it, a light lake, approaching vermilion, from the detention of blood in its capillary system. The following experiment proved this:

June 1827, at the University.—A full-grown female rabbit, of the white kind, was fed upon green cabbage leaves. In about three hours afterwards she was killed, by pithing with a saddler's awl between the occiput and the first vertebra. Immediately upon the introduction of the instrument, the body was seized with a spasm, the extremities were drawn together and convulsed; the animal gaped two or three times, but there was no inspiration. In five minutes after pithing, the abdomen was opened: the circulation was found, by pressure upon the veins, to be going on but languidly in the parietes of the abdomen and in the abdominal viscera. I waited five minutes more, when it seemed to be completely stopped.

I then cut out the stomach, laid it open along its great curvature, and washed away with a stream of water its contents, which presented the appearance of having been well boiled and bruised. The food would have measured probably about an ounce, and was principally in the left half of the stomach: there was no hour-glass contraction of the latter. The mucous coat of the left half of the stomach was of a uniform light lake, approaching vermilion, without blotches or shades of difference, and firm; but the mucous coat of the right half was almost destitute of vessels or injection, and had the dull pearl colour of the stomachs of the sheep bled to death. The mucous coat of the small intestines was the same colour as the stomach, but much lighter.

The vermicular motion continued for ten or fifteen minutes in the intestines, after the abdomen was opened. I observed that the same occurred in the horns and body of the uterus, quite as clearly as in the intestines, and that it could be produced at pleasure by puncture, by clipping, and by stirring. On the uterus being slit open from one end to the other, it flattened itself out, and the internal surface corrugated precisely as in the small intestine. The capillary system of the liver bled freely half an hour after death, upon being punctured with the scissors.

From the great number of blood-vessels distributed through mucous membranes, they are, during life, of a very bright red colour, on many of the viscera, as the stomach, the small intestines, the lower end of the large, on the vagina and nose; in many other parts they are much less vascular, as in the lining membrane of the sinuses of the nose, in the bladder, and in the excretory ducts generally. The nostril and the vagina, in a robust healthy person, will probably be found to represent correctly the shade of colour which, in life, belongs naturally to the gastro-intestinal mucous coat.

*Observation 1st. Living colour of healthy mucous coat of colon.*—There is now a female in the surgical ward of the Philadelphia Alms-house, who suffered some time ago from prolapsus ani, which is said to have protruded about six inches: the protruded intestine sloughed off, as well as the sphincter ani and the adjoining integuments. This new state of the parts affords a distinct view of the internal coat of the colon, near the sigmoid flexure. The perineum has cicatrised and united to the end of the colon, but the surface is kept excoriated by the continued excrementitious discharges, from the want of a sphincter. In this patient, the mucous coat is of the colour of the vagina, or of a recently blistered surface of the true skin.

*Observation 2d. Sudden death from ossification of the coronary arteries, exhibiting natural colour of gastro-intestinal mucous membrane.*—July 17, 1827. Mr. W. J., aged fifty-five, of remarkably temperate, regular habits, for some years previous to his death was troubled with a shortness of breathing upon ascending a flight of stairs. His principal treatment was regularity and abstemiousness. Last night, about ten o'clock, just before he went to bed, he took, as was not unusual, a glass of cream, which he always considered to agree remarkably well with him. About four o'clock A.M. he was seized with extreme difficulty of breathing, complained of most violent pain in his loins, said he was dying, and in a short time expired. From the commencement to its termination, this attack occupied twenty minutes, during which his senses remained unimpaired. Dr. Edward Jenner Coxe was present; he found his pulse regular, but small.

At four o'clock this afternoon I examined him, assisted by Drs. Dewees and Coxe.

*Abdomen.*—Liver healthy. Spleen, coats of, interspersed with white patches, giving it a parti-coloured look; healthy in other respects.

Stomach middle sized; contained two ounces of mucus, some of which was loose, while the other formed a white transparent coat adhering to the sides, but which could be scraped off easily. Rugæ of mucous coat elevated, but not unusually so; whole internal surface of mucous coat on the summits and sides of rugæ of a very light, warm sienna, or bright brown colour, which was produced by innumerable microscopical points of red blood remaining in the capillaries. In the depressions between the rugæ, the stomach of a dull pearl colour.

The jejunum was of a deeper sienna, and of a more uniform tinge, than the stomach; contained feculent matter, consisting of mucus, yellow bile, and cream, all reduced to a homogeneous and digested mass. Its lacteals were distended with chyle, and were seen converging in their ramifications to the mesenteric trunks, which were also filled. The lacteals were not perceptible in the ileum.

The colon presented its mucous coat of a dull pearl colour, and contained soft healthy fæces adhering to its sides.

Peritoneum entirely and unexceptionably healthy.

We have now ascertained the colour of a healthy gastro-intestinal mucous coat, in death from hemorrhage after fasting; in death from puncture of medulla spinalis with a full stomach; in sudden death without the least probability of a diseased digestive apparatus, the stomach being empty; and finally in the living state. We shall next endeavour to ascertain the colour in Congestion.

### II. *Colour of Mucous Membranes from Congestion.*

Our materials for elucidating the laws and phenomena of passive congestion are exceedingly imperfect and scanty. According to one writer,* congestion is the effect of torpor of habit, connected with an abstraction of stimulus, and is for the most part local. It is seldom accompanied with febrile irritation till it acts as an extraneous stimulus. Febrile motions may sometimes be the product of congestions;—congestions, depositions, or evacuations, are uniformly the product of febrile motions, that is, of irregular action. Dr. Armstrong has also written, indeed diffusely, on what he calls the congestive typhus fever, (p. 68—97;) but the cases which he has given in illustration of his views would seem rather to belong to diseases of irritation, than to passive congestion of

* Jackson on Fever; 1803, p. 197.

the organs affected. He has also made some capital omissions in not giving us the laws of congestion,—in not saying a word about the state of the mucous coat of the stomach and bowels,—and in confining his attention almost exclusively to the liver, spleen, and brain.

The congestion of red blood in any part of the body is commonly produced by an obstruction of one or more of the large venous trunks which return the blood to the heart. A ligature on the arm for the purpose of bleeding has this effect; garters worn too tightly, or indeed any other articles of dress which interfere with the return of blood, produce the same; lymphatic or aneurismal tumors situated near the junction of the extremities with the trunk of the body, also produce a congestion of blood in the parts beyond them, by pressing on the adjoining veins. Position will do the same, as throwing the head downwards and forwards in certain individuals who are corpulent and have short necks. Permitting a column of blood to remain too long on a part sometimes has this effect, as in standing or walking, which in certain persons is followed by a great accumulation of blood in the legs and feet. The general character, however, of all these cases of congestion is, that the congestion disappears the moment that the cause that produced it is removed. It very rarely happens that the capillaries and the larger veins are more than simply distended under such circumstances, and they quickly contract to their healthy diameters. It, however, occurs occasionally in pregnant women, near the term, to have some of the fine blood-vessels of the legs ruptured, in which case there follows an extravasation of blood in small bluish spots, about half an inch in diameter, and which, being a true ecchymosis, presents the same variations of colour, and is removed by the same process as the latter when caused by bleeding.

It is practicable to produce a congestion of the whole venous system, by obstructing the general circulation. If such obstruction be continued till the death of the individual, the phenomena of congestion are exhibited in swollen face, bulging eyes, blue lips, tumefied and red tongue, with purplish or red blotches on various parts of the surface of the body, but especially about the head, neck, and trunk. The most usual causes of such obstructions to the general circulation are asphyxia, from hanging, drowning, or irrespirable gases. The mucous membrane of the lungs wanting in this case the usual stimulus of atmospheric air, refuses passage to the blood from the ramifications of the pulmonary artery into those of the veins, and a complete arrest is thus put upon the general circulation. The lung becomes the seat of a congestion or engorgement in its capillary system, and all the mucous membranes of the body suffer in the same way.

Bichat's experiments* are quite conclusive on this point. He says, that in an animal in whose trachea a stop-cock has been fixed, if you draw out a portion of intestine and split it open, then close the stop-cock, in four or five minutes afterwards a dark brown colour will succeed to the red, which previously characterised the mucous surface of the gut; and the same change of colour will occur in a granulating surface under similar circumstances.

The lividity of the surface of the body, and of its mucous membranes, may be made to come and go at pleasure, by interrupting or restoring the freedom of respiration. The arrest of the capillary circulation is, however, not to be considered as a mechanical phenomenon, but a physiological one; for the animalization of the blood being somewhat affected or altered from the want of respiration, the sensibility of the capillary system is not properly excited by it, and therefore this system refuses to propel the blood. The blood in this respect may be compared to extraneous fluids, which, if they be injected into living blood-vessels, are found not to run so minutely as they do some time after death, when the vessels get into a passive state.† Parallel phenomena are common, and are well exhibited in the actions and sensibilities of many parts of the body. For example, the trachea, which is so large, yet closes immediately against the introduction of fluids which are unfit for respiration, as proved by Goodwin's experiments. The urethra, in a state of sexual excitement, will transmit the semen, but not the urine; the lacteals will readily absorb the nutritious parts of the chyle, but refuse the remainder, notwithstanding the equal fluidity of both. A moment's reflection will satisfy us that such powers of discrimination are indispensable to the operations of the system; for without them the several fine tubes of the human body, vascular, absorbent, and secretory, would be continually mixing their fluids in a manner inconsistent with life and its objects.

It is not improbable that several species of purpura, as the urticans, the senilis, and the hæmorrhagica, all of which seem to be an arrest of the red blood in patches in the true skin, or rete mucosum, have, in their pathology, a close connexion with the purple spots from asphyxia: that is to say, that there is a want of reciprocal sympathy between the red blood and the capillaries of the part, and therefore it is arrested in its passage

* Anat. Gen. vol. i. p. 188. † Ib. vol. ii. p. 25.

through the latter. These spots may be distinguished from a common ecchymosis by their boundaries being well defined, and also by their deep, varying colour, which being sometimes lighter and sometimes darker alternately, show that the blood forming them, though arrested, is yet under the influence of animalization. The colour alternating is, however, not an invariable feature of this disease. I have lately met with a case of purpura hæmorrhagica at the Alms-house following an inflammation in the cellular substance on the front of the knee, and extending over the anterior semi-diameter of the leg from the knee to the foot, where there were no alternations of colour, and the disease disappeared like a common ecchymosis. I have subsequently seen it a prelude to mortification in the leg of a patient, Anthony Hartman, aged twenty-five, in the same institution. The patch which it formed surrounded almost the whole leg about its middle, in a band of five or six inches.

In the more common cases of congestion, as the accumulation of blood is removed immediately upon the cause being withdrawn, so the organ of the body which has been the seat of it is quickly restored to its usual functions, and the disturbance which they have experienced readily disappears. If, however, the congestion be permitted to remain an undue length of time, the preponderance of the fluids will cause the congested part to inflame, and even to mortify, as in strangulated hernia.

The following dissection of a patient who died in the Alms-house will serve to illustrate the state of the stomach and bowels in congestion from a slow species of asphyxia, and to fill up partially the very wide gap in this part of physiology and pathology.

*Observation 3d. Congestion of gastro-intestinal mucous coat.*—W. T. ætat. thirty-seven, admitted June 8th, 1827, with abscess round cricoid cartilage; died June 10th.

*Abdomen*: System of vena portarum filled with blood, even in the fine intestinal branches, which were very conspicuous under peritoneum, so as to give a light purple colour to the whole of the intestinal canal; in places it was interspersed with mahogany coloured patches of two inches diameter. Colon externally was of a white pearl colour, contracted to three-eighths of an inch in diameter, and contained natural hard fæces. There were no fæces in small intestines, only flatus.

Along the whole anterior margin of the liver, a little above its edge, there was a white hard cicatrix of old condensed coagulating lymph, mixed at intervals with hepatic structure, by penetrating at various places from three to six lines into it. This terminated near the right end of the great lobe, by a depression, as if an abscess had once existed there. On cutting into the liver, near the cicatrix, many small miliary tubercles were found there, but they were not observed at more remote places.

Gall-bladder filled with healthy bile.

Spleen healthy, but completely surrounded by old adhesions.

Stomach, common size; of a pearl colour on peritoneal surface. Internal or mucous coat covered with mucus tinged with bile, corrugated, light pink colour generally; summit of rugæ, from accumulation in capillaries, appeared like red streaks, at the distance of six feet, from being covered with minute dots of red of the size of a needle-point. No large patches of ecchymosis or red blotches, so conspicuous in inflammation.

Intestines: mucous coat covered with similar small dots or points of red. Internal membrane of colon slightly tinged with red dots. Besides the hardened fæces in the commencement of this viscus, there were some small pieces of it along its course.

Œsophagus natural; internal coat, white pearl colour, and the venous ramuscules filled with blood, but the dots not conspicuous.

*Throat:* Along the upper margin of the glottis, as formed by the doubling of the membrane from the tip of the arytenoid to the side of the epiglottis, a tumor existed on each side, formed of serum and coagulable lymph, about the size of a small nutmeg, so loose as to hang over the glottis, and to be drawn over the rima glottidis in every act of inspiration.

To sum up what has been said, it appears then—

1st. That congestion is not an active condition of the part affected. When active, it constitutes inflammation.

2d. That congestion most frequently is the result of mechanical impediments to the venous circulation.

3d. That the other cases in which it occurs are where there is a want of reciprocal sympathy between the blood and the blood-vessels in the capillary system, in consequence of which the latter refuses passage to the red blood.

